# Dietary total antioxidant capacity interacts with a variant of chromosome 5q13-14 locus to influence cardio-metabolic risk factors among obese adults

**DOI:** 10.1186/s43042-022-00328-3

**Published:** 2022-08-05

**Authors:** Mahdieh Khodarahmi, Amir Sobhrakhshan Khah, Mahdieh Abbasalizad Farhangi, Goli Siri, Houman Kahroba

**Affiliations:** 1 Department of Community Nutrition, Faculty of Nutrition and Food Science, Tabriz University of Medical Sciences, Tabriz, Iran; 2 Sepehr Heart Center, Baharloo Hospital, Tehran University of Medical Sciences, Tehran, Iran; 3 Drug Applied Research Center, Tabriz University of Medical Sciences, Attar-neishabouri Ave, Golgasht St, Tabriz, 5165665931 Iran; 4 Department of Internal Medicine, Amir-Alam Hospital, Tehran University of Medical Sciences, Tehran, Iran; 5 Department of Toxicogenomics, GROW School of Oncology and Development Biology, Maastricht University, Maastricht, The Netherlands; 6 Centre for Environmental Sciences, Hasselt University, Hasselt, Belgium

**Keywords:** Obesity, CARTPT, Gene–diet interaction, Polymorphism, Total antioxidant capacity, Metabolic factors

## Abstract

**Background:**

The association between cocaine- and amphetamine-regulated transcript prepropeptide gene (CARTPT) and obesity-related outcomes has shown in the epidemiological studies. Nevertheless, there is lack of data regarding the CARTPT gene–diet interactions in terms of antioxidant potential of diet. So, this study aimed to test CARTPT gene–dietary non-enzymatic antioxidant capacity (NEAC) interactions on cardio-metabolic risk factors in obese individuals.

**Methods and material:**

The present cross-sectional study was carried out among 288 apparently healthy obese adults within age range of 20–50 years. Antioxidant capacity of diet was estimated by calculating the oxygen radical absorbance capacity (ORAC), ferric reducing antioxidant power (FRAP), total radical-trapping antioxidant parameter (TRAP) and Trolox equivalent antioxidant capacity (TEAC) using a semiquantitative food frequency questionnaire (FFQ). Genotyping for CARTPT rs2239670 polymorphism was conducted by polymerase chain reaction–restriction fragment length polymorphism (PCR–RFLP) method.

**Results:**

A significant interaction was revealed between CARTPT rs2239670 and dietary ORAC on BMI (*P*_Interaction_ = 0.048) and fat mass percent (FM%) (*P*_Interaction_ = 0.008); in A allele carriers, higher adherence to the dietary ORAC was related to lower level of BMI and FM%. And, the significant interactions were observed between FRAP index and rs2239670 in relation to HOMA (*P*_Interaction_ = 0.049) and QUICKI (*P*_Interaction_ = 0.048). Moreover, there were significant interactions of rs2239670 with TRAP (*P*_Interaction_ = 0.029) and TEAC (*P*_Interaction_ = 0.034) on the serum glucose level; individuals with AG genotype were more respondent to higher intake of TRAP.

**Conclusion:**

The present study indicated that the relationships between CARTPT rs2239670 and obesity and its-related metabolic parameters depend on adherence to the dietary NEAC. Large prospective studies are needed to confirm our findings.

## Introduction

Obesity, as a worldwide pandemic, has become a major public health issue, and its prevalence is increasing markedly in many countries [1]. In comparison with 1980, global prevalence of overweight and obesity has nearly doubled in all age–sex groups regardless of race, ethnicity and socioeconomic status [2]. Obesity is a multifactorial and preventable disorder which substantially increases risk of comorbidities such as: cardiovascular diseases (CVDs), stroke, type 2 diabetes, hypertension, fatty liver disease and certain cancers. Thereby, it can affect quality of life, life expectancy, work productivity and healthcare costs [1]. A wealth of evidence has shown that obesity contributes to initiation and progression of these pathological conditions through oxidative stress which is defined as an imbalance between reactive oxygen species (ROS) production and antioxidant defenses [3]. It has been demonstrated that oxidative stress damages or alterations in antioxidant defenses are involved in the pathogenesis and development of obesity-associated consequences such as CVDs and cancer [3–5].

Emerging evidence suggests that the development of obesity, as a multifactorial disorder, and its-related comorbidities is determined by interactions between genetic and environmental variables particularly diet [6]. Accumulating evidence suggests that dietary antioxidant intake (e.g., selenium, vitamin C and vitamin E) through non-enzymatic defense mechanisms can protect against cell damage caused by oxidative stress, and its-related inflammatory outcomes [7]. In this regard, the epidemiological research has reported that higher intakes of fruits and vegetables, which are rich sources of antioxidants, are associated with the lower risk of several chronic conditions such as CVDs and cancers [8, 9]. Since various antioxidants are combined in foods and they act in a cumulative and synergistic way in complex matrixes, assessment of single specific antioxidant may not reflect the potential overall antioxidant effect of the diet. Thus, investigation of the dietary total antioxidant capacity (TAC) can provide a better method to examine the favorable influences of dietary antioxidants on chronic diseases prevention [10]. Non-enzymatic antioxidant capacity (NEAC), also recognized as TAC, is a new approach to capture synergistic effects of antioxidants in diet and generally can be estimated through four different methods including: Trolox equivalent antioxidant capacity (TEAC), ferric reducing antioxidant power (FRAP), total radical-trapping antioxidant parameter (TRAP) and oxygen radical absorbance capacity (ORAC) [11]. Recently, evidence from observational investigations has revealed inverse significant associations of dietary NEAC with the risk of stroke, diabetes and different cancers [12–14].

**Fig. 1:**
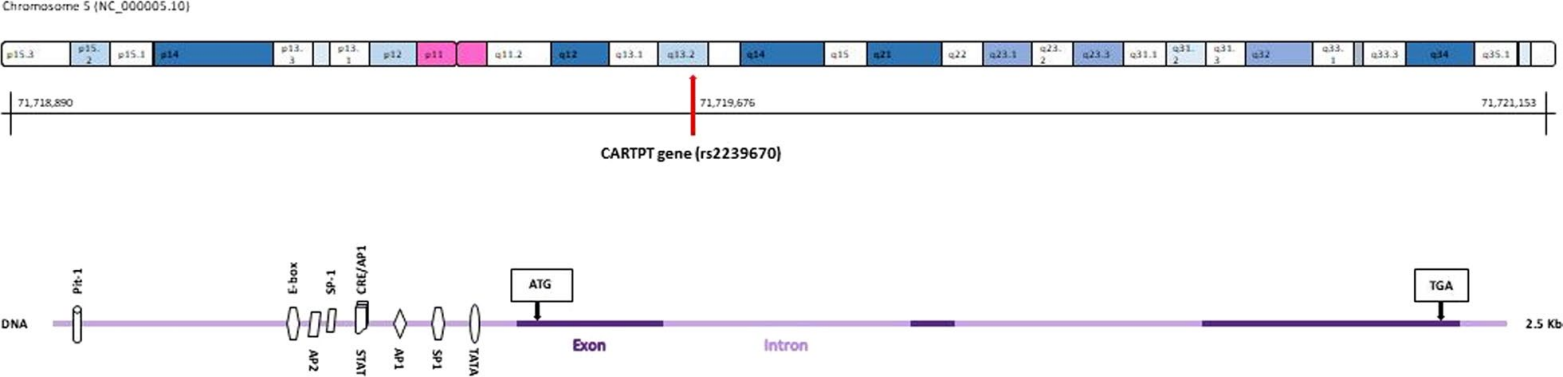
Overview of genomic structure of the CARTPT gene and location of rs2239670 polymorphism

Since obesity is highly heritable, genetic factors substantially play an important role in development of obesity and its serious consequences [15]. Cocaine- and amphetamine-regulated transcript prepropeptide gene (CARTPT), which maps to the chromosome 5q13-14 and expresses the cocaine- and amphetamine-regulated transcript (CART) protein, has been recognized to be a susceptibility locus for obesity (Fig. 1). CART protein, as one of the various neuropeptides in the arcuate nucleus (ARC) of the hypothalamus, has been implicated in modulating feeding behavior and energy balance [16]. In this regard, previous studies have found that CART peptides which are co-expressed with other neurotransmitters such as α-melanocyte-stimulating-hormone (α-MSH) inhibit food intake [17]. The latest scientific studies have indicated that variations in the CARTPT gene might influence obesity, metabolic syndromes (MetS) and its components [18]. However, the results of studies regarding these associations are controversial [19]. These heterogeneous associations may be due to the complicated pathogenesis and etiology of obesity which involve interactions between genetic and environmental factors especially diet. A positive association between CARTPT rs2239670 variant and alcoholism has been reported in the Korean population [20]. Since obesity and substance abuse have a common neurobiological basis, investigation of the association between this polymorphism and obesity and metabolic factors is interesting.

Therefore, since the better understanding of the gene–diet interactions can provide more efficient strategies for personalized treatment, and also as far as we know, no previous evidence is available on the modification effect of diet in terms of dietary antioxidant capacity on the association of CARTPT gene with obesity-related metabolic factors, the aim of the present study was to assess the potential interactions of CARTPT rs2239670 with NEAC intake in relation to cardio-metabolic risk factors among obese population.

## Materials and methods

### Study participants

This cross-sectional study was carried out in Tabriz, a city in the northwest of Iran, from December 2017 to April 2019 among apparently healthy obese subjects using convenience sampling method. Study participants were 288 apparently healthy obese (body mass index (BMI) ≥ 30 kg/m^2^) adult aged 20–50 years who were obese. Detailed illustration of recruitment of research participants has been provided elsewhere [21]. Individuals were removed from the study if they met any of the following criteria: being menopausal and pregnant, lactation, prior diagnose of diseases (cardiovascular diseases, hypertension, hyperlipidemia, cancer, type 2 diabetes mellitus and renal diseases), taking any medication affecting the variables studied (hypoglycemic, lipid-lowering, antihypertensive, corticosteroids or antidepressants). All subjects completed a written, informed consent and the ethical committee of Tabriz University of Medical Sciences approved the protocol of this study (Ethics number: IR.TBZMED.REC.1397.266).

### Demographic and anthropometric assessments

Information of physical activity and other demographic characteristics (age, sex) was collected by trained interviewer at the beginning of the study. To estimate the level of physical activity, the short form of the international physical activity questionnaire (IPAQ) was used [22]. Weight and height were measured in light clothing using a Seca scale (Seca, Germany) and a tape measure to the nearest 0.1 kg and 0.1 cm, respectively. Waist and hip circumference (WC and HC) was obtained at the narrowest level and largest part, respectively, by a non-elastic measuring tape with accuracy of 0.1 cm, and waist-to-hip ratio (WHR) was then calculated. Assessments of body composition were conducted using bioelectrical impedance analysis (BIA) technology (Tanita, BC-418 MA, Tokyo, Japan). Blood pressure of subjects was determined using a mercury sphygmomanometer after the participants had a 10-min rest in a sitting position. This measurement was carried out two times, and the average of them was considered as subject’s blood pressure.

### Biochemical measurements

Blood samples were taken from all study participants after a 12-h fasting period. Plasma and serum were prepared by centrifugation (10 min at 4500 rpm, 4 °C), and their aliquots were frozen at − 80 °C until they were analyzed. Analyses of serum glucose, total cholesterol (TC), triglyceride (TG) and high-density lipoprotein cholesterol (HDL-C) levels were conducted by commercially available kits (Pars Azmoon Inc., Tehran, Iran) [21]. Serum insulin [23] and plasma concentrations of α-MSH and AgRP were measured by enzyme-linked immunosorbent assay kits (Bioassay Technology Laboratory, Shanghai Korean Biotech, Shanghai City, China) [24] based on manufacturer’s protocol. Serum low-density lipoprotein cholesterol (LDL), the homeostasis model of assessment ratio (HOMA-R) and quantitative insulin sensitivity check index (QUICKI) were calculated using the protocols which have been described by Friedewald et al. [25], Matthews et al. [26] and Katz et al. [27], respectively.

### Assessment of dietary intakes and NEAC calculation

Dietary intake of participants during the previous year was evaluated using a 147-item semiquantitative food frequency questionnaire (FFQ) which was previously validated for use among Iranian population [28, 29]. Study subjects were asked to report portion size of each food item during the previous year on a daily, weekly, monthly and yearly basis. By using household measurements, portion sizes of consumed food items were converted to grams [30]. Then, Iranian Food Composition Table (FCT) was applied to analyze daily nutrient intake [31]. Information missing from this FCT was completed by the United States Department of Agriculture FCT [32].

NEAC is an indicator of diet quality which describes the ability of different antioxidants in food to remove free radicals, and its value was measured using the following indices: FRAP [33] which estimates the reducing potency of dietary antioxidants, TRAP [34] that evaluates the chain-breaking antioxidant potential for scavenging peroxyl radicals, ORAC [35] which measures the antioxidant capacity against peroxyl radicals using an area under curve (AUC) technique and TEAC [34] which is based on scavenging ability of antioxidants against a radical cation in both lipophilic and hydrophilic environments. Since Maillard products from the coffee roasting process are the main contributors to the high in vitro antioxidant capacity of coffee [36] and also due to high molecular weight of these products, the proportion absorbed through the intestinal mucosa and whether they display an antioxidant effect in vivo is still unknown [37], we decided to exclude the contribution of coffee to NEAC. Dietary NEAC values for 64, 63, 59 and 65 food items in the FFQ were assigned by ORAC, FRAP TRAP and TEAC, respectively. Finally, to obtain total dietary NEAC for each participant, corresponding NEAC values of single foods were multiplied by the daily intake of each food consumed and then summed up.

### DNA extraction and genotyping

The genomic DNA was extracted from blood samples by phenol/chloroform extraction method. Nano Drop 2000C spectrophotometer was applied to determine the quality and quantity of the DNA extracted from each sample. Genotyping the CARTPT rs2239670 variant was carried out by polymerase chain reaction–restriction fragment length polymorphism (PCR–RFLP) method. The following primers were used for PCR amplification: forward 5′-CCTGCTGCTGATGCTACCTCT-3′ and reverse 5′-GCGCTTCGATCTGCAACACAC-3′. The cycling conditions in DNA thermocycler were as follows: 94 °C for 5 min (initial denaturation), 35 cycles of denaturation at 94 °C for 30 s, annealing at 60 °C for 30 s and extension at 72 °C for 20 s. At the end of the final cycle, an additional extension step occurred at 72 °C for 10 min. PCR amplification was optimized in a total volume of 25 μl containing 2 µl genomic DNA, 12.5 µl distilled water, 0.5 µl of each primer and 10 µl Taq DNA Polymerase Master Mix (Ampliqon, Denmark). PCR product was digested with ApaI restriction enzyme (Takara, Japan), and then, digested product was subjected to electrophoresis on 3% agarose gel. After visualizing by electrophoresis, the A allele appeared as fragment with length of 552 bp, while G allele was distinguished as 340 and 212 bp fragments.

### Statistical analyses

All variables were checked for normality of distribution by Kolmogorov–Smirnov test. The comparison of qualitative and quantitative variables was conducted by chi- square test and analysis of variance (ANOVA), respectively. Data on continuous and categorical variables were presented as the mean ± SD and the frequencies or percentages, respectively. ANCOVA multivariate interaction model with adjustment for confounders was applied to assess the interactions between CARTPT rs2239670 polymorphism and dietary NEAC on cardio-metabolic risk factors. Statistical Package for Social Science (SPSS Inc., Chicago IL, USA) version 22.0 was employed for data analyses. A p value less than 0.05 was considered significant.

## Results

**Fig. 2:**
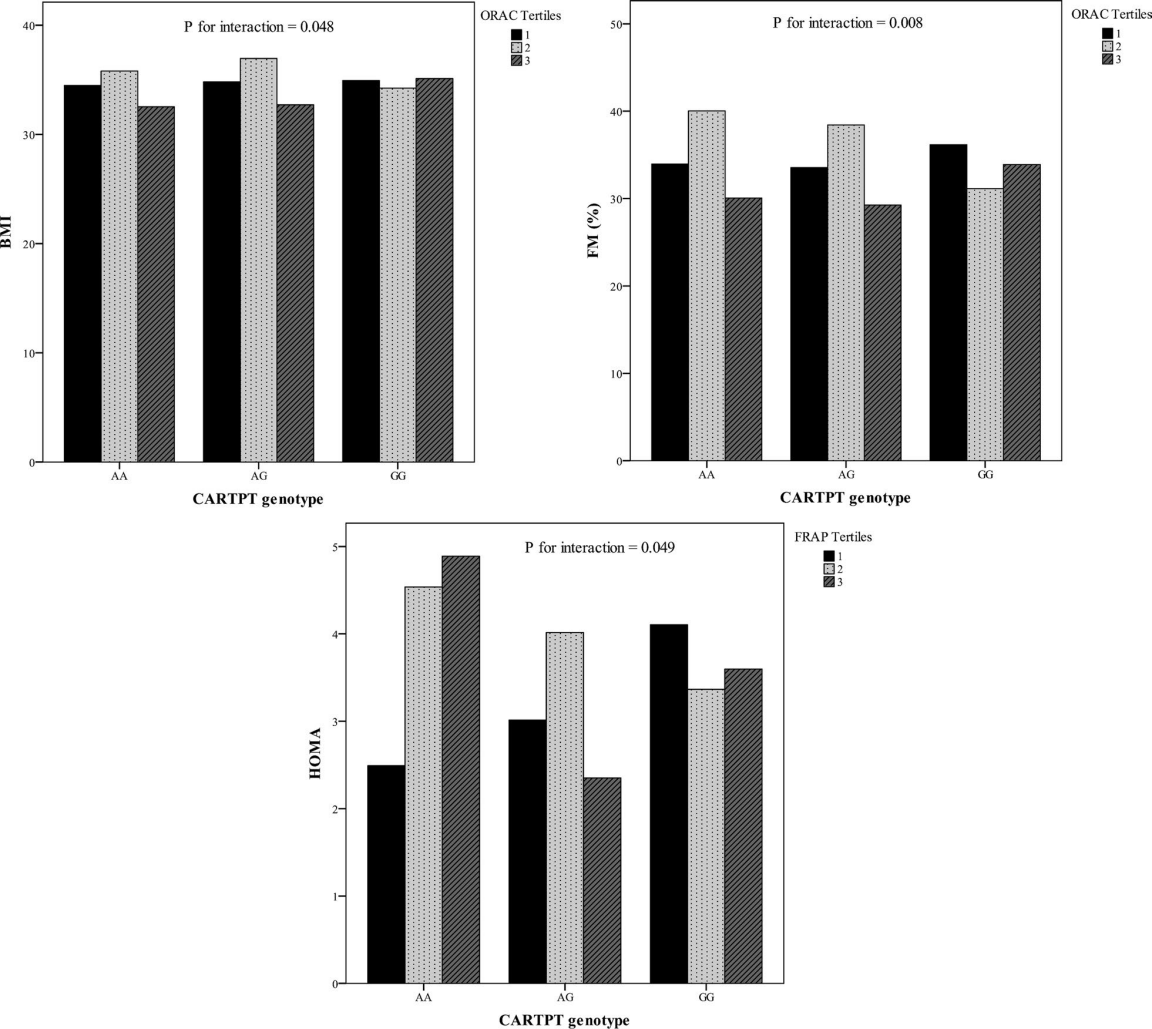
Significant interactions between dietary NEAC (ORAC and FRAP) and rs2239670 genotypes in relation to fat mass (FM), body mass index (BMI) and the homeostasis model of assessment ratio (HOMA-R)

**Fig. 3:**
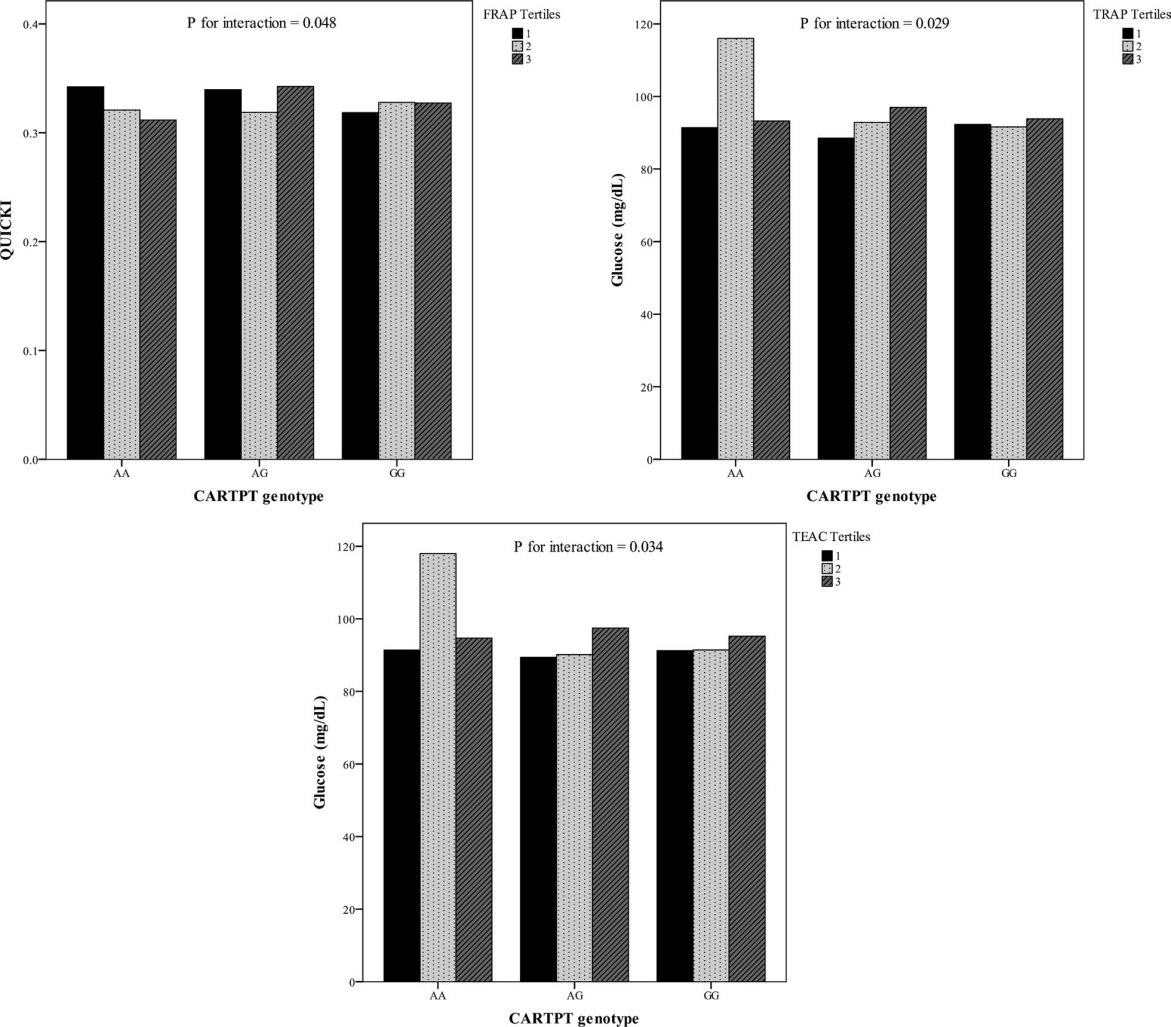
Significant interactions between dietary NEAC (FRAP, TRAP and TEAC) and rs2239670 genotypes in relation to the quantitative insulin sensitivity check index (QUICKI) and serum glucose level

The mean (SD) age and BMI of the study subjects were 38.04 (7.47) years and 34.72 (3.88) kg/m^2^, respectively. Distribution of study subjects in terms of general characteristics across CARTPT rs2239670 genotypes is shown in Table 1. A statistically significant difference was found in the mean WHR (*P* = 0.025) according to different genotypes. The analysis did not reveal any significant differences for other general characteristics. Table 2 summarizes the dietary macro- and micronutrients intakes of the study participants. The mean (± SD) values of energy, protein, carbohydrate and fats intakes were 3042.91 (1077.89), 97.86 (34.67), 440.02 (164.76) and 108.04 (46.85), respectively. The genotype and allele frequencies for the CARTPT rs2239670 polymorphism among dietary NEAC tertiles are presented in Table 3. The results of the comparison showed that participants carrying the heterozygote genotype were more likely to have lower adherence to dietary FRAP, TRAP and TEAC; however, there was no statistically significant difference. Besides, in spite of non-significant associations, minor allele carriers were assigned to the second tertile of ORAC. On the other hand, as indicated in Table 3, the frequency of mutant allele increased when following a healthy diet rich in antioxidants, while differences were not significant (*P* > 0.05). The total frequencies of genotypes among population studied were as follows: AA (10.76%), AG (20.13%) and GG (69.9%). And, the minor allele frequency observed in this study was 20.79%. There was no significant difference regarding the mean values of biochemical parameters of subjects across different genotypes of CARTPT rs2239670 variant (Table 4). As shown in Table 5, there were significant differences regarding WC, BMR, TG, LDL-C and glucose between various tertile of dietary NEAC (*P* < 0.05). Although the participants in the highest tertile of TEAC had higher means of WC (*P* = 0.031) and TG (*P* = 0.037), mean of serum concentration of LDL-C was partially lower in this category than the first tertile (*P* = 0.053). Moreover, those in the highest tertile of the NEAC (ORAC, FRAP, TRAP and TEAC) had higher level of BMR (*P* < 0.05) than those in the lowest category. Similarly, higher means of glucose level (*P* = 0.018) were seen among those in third tertile of the ORAC. In addition, participants who assigned to the second tertile of FRAP had higher WC (*P* = 0.04) compared to other tertiles. As elucidated in Fig. 2, after adjusting for sex and age, significant interactions were revealed between CARTPT rs2239670 variant and dietary ORAC on BMI (*P*_Interaction_ = 0.048) and fat mass percent (FM%) (*P*_Interaction_ = 0.008); the lowest means of BMI and FM% were observed in A allele carriers in the third tertile of dietary ORAC. Moreover, significant interactions were observed between FRAP indicator and CARTPT rs2239670 polymorphism in relation to HOMA (*P*_Interaction_ = 0.049) (Fig. 2) and QUICKI (*P*_Interaction_ = 0.048) (Fig. 3), such that adherence to the dietary FRAP modified positively the association of the CARTPT rs2239670 variant with HOMA and QUICKI in G allele carriers. In other words, dietary NEAC could not modulate detrimental effects of CARTPT rs2239670 polymorphism on insulin resistance indices (HOMA and QUICKI) in participants carrying AA genotype. Additionally, significant interactions were observed between CARTPT rs2239670 variant and TRAP (*P*_Interaction_ = 0.029) and TEAC (*P*_Interaction_ = 0.034) in relation to serum glucose level (Fig. 3); the highest serum glucose concentration was found in the AA homozygote carriers assigned to the second tertile of TRAP and TEAC.

**Table 1:** General characteristics of study subjects based on CARTPT rs2239670 polymorphism genotypes

Variables	Genotype			P^*^
	AA	AG	GG	
Gender				
Male	8 (5.4)	30 (20.4)	109 (74.1)	0.057
Female	22 (15.6)	28 (20)	90 (64.4)	
Age (year)	37.05 (7.45)	39.78 (8.33)	37.75 (7.10)	0.288
BMI (kg/m^2^)	34.35 (3.83)	34.99 (4.61)	34.76 (3.75)	0.848
WC (cm)	105.26 (10.01)	107.82 (12.86)	109.44 (8.95)	0.204
WHR	0.89 (0.08)	0.92 (0.07)	0.93 (0.07)	**0.025**
FM (%)	34.96 (8.58)	34.13 (8.62)	33.81 (9.47)	0.875
BMR (kcal)	1749.11 (312.20)	1837.59 (367.53)	1931.75 (416.11)	0.116
PA (min/week)	1396.32 (1587.27)	1232.33 (1677.67)	2341.14 (3493.41)	0.106
SBP (mmHg)	111.58 (16.51)	117.28 (14.30)	115.90 (13.88)	0.360
DBP (mmHg)	72.95 (12.48)	78.89 (10.42)	76.25 (10.90)	0.157

*BMI* Body mass index, *WC* waist circumference, *WHR* waist-to-hip ratio, *FM* fat mass, *BMR* basal metabolic rate, *PA* physical activity, *SBP* systolic blood pressure, *DBP* diastolic blood pressure; values for gender are in number of subjects (percentage) and for all other variables are presented based on mean (SD)

^*^P values were determined using one-way ANOVA and chi-square test; Bold *P*-values are less than 0.05 and are statistically significant

**Table 2:** Daily macro- and micronutrient intakes of the study participants

variables	Mean (SD) or Median (25 and 75 percentiles)
Macronutrients	
Carbohydrate (g/day)	440.02 (164.76)
Protein (g/day)	97.86 (34.67)
Fats (g/day)	108.04 (46.85)
Energy intake (kcal/day)	3042.91 (1077.89)
Micronutrients	
Fiber (g/day)	58.03 (42.30, 92.44)
Cholesterol (g/day)	278.25 (180.83, 376.32)
SFA (g/day)	30.33 (14.54)
PUFA (g/day)	25.24 (12.40)
Linoleic (g/day)	0.05 (0.02, 0.11)
Linolenic (g/day)	1.49 (0.97, 2.10)
Minerals	
Phosphor (mg/day)	1735.37 (610.56)
Magnesium (mg/day)	526.00 (197.58)
Zinc (mg/day)	14.45 (5.42)
Copper (mg/day)	2.49 (1.18)
Calcium (mg/day)	1252.08 (523.91)
Iron (mg/day)	24.22 (11.84)
Potassium (mg/day)	4397.85 (1722.54)
Selenium (mg/day)	152.97 (60.63)
Manganese (mg/day)	9.10 (3.95)
Vitamins	
A (RAE/d)	787.95 (525.18, 1092.84)
D (µg/day)	1.75 (1.00, 2.96)
E (mg/day)	14.68 (10.35, 22.54)
K (µg/day)	204.86 (147.32, 329.06)
B1 (mg/day)	2.65 (1.13)
B2 (mg/day)	2.59 (1.03)
B3 (mg/day)	29.11 (11.11)
B6 (mg/day)	2.19 (0.87)
B9 (µg/day)	745.66 (315.38)
Biotin (µg/day)	39.07 (16.36)
C (mg/day)	157.32 (105.73, 222.20)
Other variables	
Lutein (µg/day)	2189.49 (1432.70, 3006.07)
Lycopene (µg/day)	3998.40 (2300.10, 5777.14)
β-carotene (µg/day)	4593.49 (2978.49, 6487.61)
Caffeine (mg/day)	200.02 (103.37, 295.62)

*SFA* saturated fatty acid, *PUFA* poly unsaturated fatty acid

**Table 3:** Allele frequency and overall genotype prevalence for CARTPT rs2239670 polymorphism

	Genotype prevalence			Allele frequency	
	AA	AG	GG	A	G
ORAC					
Tertile 1	10% (*n* = 10)	18.3% (*n* = 18)	71.7% (*n* = 69)	6.47%	27.25%
Tertile 2	11.7% (*n* = 11)	23.3% (*n* = 23)	65% (*n* = 63)	7.86%	25.84%
Tertile 3	10.3% (*n* = 10)	19% (*n* = 18)	70.7% (*n* = 66)	6.46%	26.12%
FRAP					
Tertile 1	8.1% (*n* = 8)	22.6% (*n* = 23)	69.4% (*n* = 69)	6.72%	28.09%
Tertile 2	8.8% (*n* = 8)	15.8% (*n* = 14)	75.4% (*n* = 70)	5.36%	26.68%
Tertile 3	15.3% (*n* = 14)	22% (*n* = 21)	62.7% (*n* = 60)	8.71%	24.44%
TRAP					
Tertile 1	8.2% (*n* = 8)	24.6% (*n* = 24)	67.2% (*n* = 67)	7.02%	27.25%
Tertile 2	10.7% (*n* = 10)	12.5% (*n* = 11)	78.8% (*n* = 69)	5.36%	26.12%
Tertile 3	13.1% (*n* = 13)	23% (*n* = 23)	63.9% (*n* = 63)	8.41%	25.84%
TEAC					
Tertile 1	8.3% (*n* = 8)	25% (*n* = 24)	66.7% (*n* = 65)	7.02%	26.68%
Tertile 2	8.8% (*n* = 8)	12.3% (*n* = 11)	78.9% (*n* = 73)	4.78%	27.25%
Tertile 3	14.8% (*n* = 15)	23% (*n* = 32)	62.3% (*n* = 61)	8.99%	25.28%
Total	10.76% (*n* = 31)	20.14% (*n* = 58)	69.10% (*n* = 199)	20.79%	79.21%

*ORAC* oxygen radical absorbance capacity, *FRAP* ferric reducing antioxidant power, *TRAP* total radical-trapping antioxidant parameter, *TEAC* Trolox equivalent antioxidant capacity

**Table 4:** The associations of CARTPT rs2239670 polymorphism and biochemical parameters

Variables	Genotype			P^*^
	AA	AG	GG	
TC (mg/dL)	192.95 (32.75)	185.94 (32.65)	188.37 (34.48)	**0.768**
TG (mg/dL)	115.26 (45.90)	109.50 (54.61)	122.34 (59.70)	**0.478**
HDL-C (mg/dL)	46.32 (9.49)	44.64 (9.12)	44.58 (8.25)	**0.709**
LDL-C (mg/dL)	123.57 (27.51)	119.40 (31.98)	119.32 (31.60)	**0.856**
Glucose (mg/dL)	99.95 (22.69)	92.67 (20.11)	92.53 (11.76)	**0.137**
Insulin (µIU/mL)	17.16 (10.57)	12.81 (6.74)	16.14 (8.61)	**0.085**
HOMA-IR	4.16 (2.62)	3.02 (1.84)	3.69 (2.02)	**0.109**
QUICKI	0.32 (0.03)	0.33 (0.03)	0.32 (0.02)	**0.110**
α-MSH (ng/mL)	2.32 (0.31)	2.29 (0.24)	2.23 (0.20)	**0.137**
AgRP (pg/mL)	1.45 (0.27)	1.46 (0.19)	1.42 (0.18)	**0.448**

*TC* Total cholesterol, *TG* triglyceride, *HDL-C* high-density lipoprotein cholesterol, *LDL-C1* low-density lipoprotein cholesterol, *HOMA-IR* Homeostatic Model Assessment for Insulin Resistance, *QUICKI* quantitative insulin sensitivity check index, *α-MSH* alpha-melanocyte-stimulating hormone, *AgRP* agouti-related peptide

^*^Using one-way ANOVA; Bold *P*-values are less than 0.05 and are statistically significant

**Table 5:** General characteristics and metabolic factors of study population by dietary NEAC tertiles

	Tertile 1	Tertile 2	Tertile 3	*P*
Age (year)				
ORAC	39.17 (7.37)	37.02 (7.54)	37.79 (7.17)	0.252
FRAP	37.27 (8.28)	37.46 (6.57)	39.27 (7.13)	0.248
TRAP	37.43 (8.14)	37.83 (6.97)	38.74 (7.03)	0.597
TEAC	37.79 (8.17)	37.09 (6.75)	39.13 (7.13)	0.293
WC (cm)				
ORAC	107.77 (11.06)	109.70 (9.26)	108.96 (9.46)	0.548
FRAP	106.27 (9.63)	110.51 (10.16)	109.66 (9.67)	**0.040**
TRAP	106.61 (8.53)	109.42 (11.35)	110.42 (9.49)	0.084
TEAC	107.20 (9.24)	107.76 (10.57)	111.50 (9.54)	**0.031**
FM (%)				
ORAC	35.61 (10.72)	33.45 (8.47)	32.34 (7.76)	0.126
FRAP	35.07 (9.38)	33.98 (9.13)	32.34 (8.81)	0.244
TRAP	35.10 (9.39)	33.49 (8.91)	32.82 (9.09)	0.359
TEAC	35.41 (9.49)	32.43 (8.61)	33.63 (9.18)	0.187
BMR (kcal)				
ORAC	1801.08 (329.37)	1965.95 (361.25)	1948.65 (472.04)	**0.037**
FRAP	1780.92 (345.87)	1941.42 (356.25)	1994.05 (454.69)	**0.007**
TRAP	1773.71 (336.63)	1961.78 (482.33)	1980.71 (322.61)	**0.005**
TEAC	1782.41 (347.19)	1910.27 (351.02)	2022.14 (453.44)	**0.003**
PA (min/week)				
ORAC	1453.22 (2165.09)	2635.49 (3851.55)	2400.58 (3337.42)	0.092
FRAP	1494.06 (2103.70)	2192.51 (3142.85)	2809.21 (4045.21)	0.073
TRAP	1531.54 (2076.53)	2554.37 (3665.86)	2403.44 (3621.95)	0.158
TEAC	1584.16 (2150.36)	2362.73 (3612.51)	2532.13 (3628.45)	0.217
SBP (mmHg)				
ORAC	116.27 (14.32)	117.43 (13.54)	114.68 (15.91)	0.574
FRAP	113.37 (11.98)	118.30 (17.11)	116.74 (13.97)	0.152
TRAP	115.17 (12.48)	115.83 (15.99)	117.42 (15.92)	0.678
TEAC	114.65 (12.07)	115.73 (16.13)	118.03 (15.21)	0.420
DBP (mmHg)				
ORAC	77.10 (10.45)	76.97 (11.46)	76.11 (12.08)	0.871
FRAP	74.97 (10.70)	77.11 (11.32)	78.13 (11.81)	0.280
TRAP	76.02 (11.15)	75.86 (10.84)	78.34 (11.89)	0.392
TEAC	76.24 (11.22)	75.61 (10.69)	78.37 (11.95)	0.360
TC (mg/dL)				
ORAC	189.97 (34.22)	190.62 (32.19)	184.13 (34.84)	0.499
FRAP	187.14 (33.58)	191.67 (36.56)	185.94 (30.98)	0.607
TRAP	193.78 (36.78)	184.95 (34.28)	186.02 (29.43)	0.278
TEAC	194.08 (35.08)	182.66 (33.93)	188.23 (31.57)	0.164
TG (mg/dL)				
ORAC	114.81 (51.75)	116.79 (65.47)	128.27 (56.90)	0.382
FRAP	106.67 (48.15)	123.94 (55.37)	129.29 (68.29)	0.075
TRAP	107.01 (47.46)	125.06 (58.86)	127.81 (65.96)	0.094
TEAC	106.40 (42.35)	120.16 (60.34)	133.18 (67.12)	**0.037**
HDL-C (mg/dL)				
ORAC	45.29 (9.10)	46.19 (9.66)	43.37 (7.45)	0.191
FRAP	45.44 (9.17)	45.97 (9.71)	43.44 (7.36)	0.241
TRAP	46.41 (9.61)	44.76 (8.81)	43.68 (7.89)	0.219
TEAC	46.32 (9.45)	45.39 (9.21)	43.15 (7.51)	0.119
LDL-C (mg/dL)				
ORAC	121.72 (30.52)	121.07 (28.75)	115.10 (33.33)	0.422
FRAP	120.36 (31.40)	120.91 (33.79)	116.64 (27.41)	0.705
TRAP	125.96 (33.09)	115.18 (30.82)	116.78 (27.88)	0.107
TEAC	126.47 (32.62)	113.23 (29.87)	118.44 (29.11)	**0.053**
Glucose (mg/dL)				
ORAC	91.32 (10.95)	92.11 (13.31)	101.52 (34.46)	**0.018**
FRAP	91.70 (11.37)	98.32 (33.54)	94.82 (16.11)	0.258
TRAP	91.57 (9.76)	98.56 (33.46)	94.71 (17.22)	0.220
TEAC	91.10 (20.29)	97.80 (33.01)	95.85 (17.34)	0.232
Insulin (µIU/mL)				
ORAC	16.47 (10.05)	14.51 (8.59)	16.58 (8.91)	0.365
FRAP	17.11 (10.52)	15.62 (8.54)	14.81 (8.39)	0.371
TRAP	16.05 (10.15)	16.48 (8.98)	15.01 (8.48)	0.655
TEAC	16.10 (10.20)	16.44 (8.80)	14.99 (8.63)	0.658
HOMA-IR				
ORAC	3.80 (2.59)	3.31 (2.07)	4.17 (2.78)	0.161
FRAP	3.94 (2.67)	3.82 (2.69)	3.51 (2.14)	0.623
TRAP	3.71 (2.61)	4.01 (2.72)	3.57 (2.21)	0.612
TEAC	3.68 (2.60)	3.99 (2.64)	3.60 (2.28)	0.659
QUICKI				
ORAC	0.32 (0.03)	0.33 (0.03)	0.32 (0.02)	0.111
FRAP	0.32 (0.03)	0.32 (0.02)	0.32 (0.02)	0.708
TRAP	0.32 (0.03)	0.32 (0.02)	0.32 (0.03)	0.408
TEAC	0.32 (0.03)	0.32 (0.02)	0.32 (0.02)	0.465

*WC* Waist circumference, *FM* fat mass; BMR basal metabolic rate, *PA* physical activity, *SBP* systolic blood pressure, *DBP* diastolic blood pressure, *TC* total cholesterol, *TG* triglyceride, *HDL-C* high-density lipoprotein cholesterol, *LDL-C* low-density lipoprotein cholesterol, *HOMA-IR* Homeostatic Model Assessment for Insulin Resistance, *QUICKI* quantitative insulin sensitivity check index; all data are mean (± SD)

*P* values derived from one-way ANOVA with Tukey’s post hoc comparisons; Bold *P*-values are less than 0.05 and are statistically significant

## Discussion

As far as we know, the current research is the first attempt to examine the interactions of CARTPT rs2239670 polymorphism with dietary NEAC in relation to cardio-metabolic risk factors. Our findings suggest that the rs2239670 on chromosome 5q13-14 interacts with dietary ORAC, FRAP and TEAC to influence obesity and obesity-related metabolic phenotypes. Specifically, when compliance with ORAC was high, minor allele carriers were less susceptible to the development of obesity-related traits such as BMI and FM%. On the other hand, improving the adherence to dietary FRAP might reduce the genetic association with insulin resistance indices (HOMA-IR and QUICKI) only among G allele carriers (AG and GG). However, positive interactions of CARTPT rs2239670 on serum glucose level were found even in high compliance with TRAP and TEAC. Thus, the results of modification effect of diet on the associations of CARTPT with obesity and metabolic factors were not homogenous. According to our results, frequency of the rare allele (20.79%) was nearly similar to that of the other population or ethnic groups such as Korean (17%) [20] and Malaysian (30%) [19]. This discrepancy in the minor allele frequency reported might be due to variations in sample size, demographic characteristics of population like age, ethnicity and gender and also various lifestyles.

Previous evidence has revealed that polymorphisms in the CARTPT gene are linked to human obesity [16]. In this regard, leu34Phe missense mutation in CARTPT gene was detected in Italian subjects with early-onset obesity [16]. Likewise, it was reported that the A-156G polymorphism in the promoter region of CARTPT was related to adiposity among Japanese subjects [38]. Moreover, the studies have shown that genetic polymorphisms in the CARTPT gene might affect susceptibility to MetS and its components such as dyslipidemia, high blood pressure and hyperglycemia [18]. It should be taken into account that all variants in CARTPT gene have not been related to obesity phenotypes and findings in this regard are conflicting [39]. For example, in accordance with the results of Walder study, C1442G polymorphism of CARTPT gene was not related to obesity among Pima Indians [39]. Other studies which have specifically investigated the association of the CARTPT rs2239670 polymorphism with addictive behaviors such as alcohol dependence have confirmed a positive relation [20]. However, the studies which have evaluated this variant in relation to obesity are scarce. According to our knowledge, only one study has assessed the CARTPT rs2239670–obesity relation and showed no association between this variant and obesity among the Malaysian subjects [19]. All of these heterogeneities in findings warrant further research efforts among different populations. Since the rs2239670 variant located in the intron 1 of CARTPT gene and its strong effects on obesity and related metabolic factors may be removed during splicing process of mRNA encoding CART proteins, effects of this variant on CART function are still unknown [19]. However, genetic polymorphisms in the CART locus may affect the expression of the CART peptide, which is related to hypothalamic anorectic and orexigenic neuropeptides [40]. Totally, it seems that CART peptides have a modulatory role in feeding behavior and exert anorexigenic effect, although the biological mechanism of this function remains unclear [41].

As far as we are aware, no previous study has investigated the gene–diet interactions of CARTPT with adherence to the dietary NEAC on metabolic profile in obesity to compare our finding. However, there has been a lot of research investigating the interaction between genetic variations and diet or dietary ingredients on obesity and its related complications [42]. For example, Mirzababaei et al. examined the interaction of the rs1333048 variant on 9p21 genetic region with TAC on the risk of MetS and they revealed that high ORAC intake may improve the increased risk of MetS in homozygous subjects for the minor allele (AA genotype) [42]. Subsequent study by Mahmoudi-Nezhad et al. has documented the significant interactions between healthy dietary patterns (healthy eating index and diet quality index-international (DQI-I)) and CARTPT rs2239670 genotypes affecting metabolic parameters [43]; higher compliance with DQI-I decreased the metabolic risk parameters in AA homozygote carriers.

Noticeably, the main finding of the present research was that the association of the CARTPT rs2239670 polymorphism with cardio-metabolic factors depended on the dietary antioxidant intakes; a good compliance with NEAC blunted the relationship between the CARTPT gene and cardio-metabolic risk factors. While the underlying mechanisms behind this interactions are not still clarified, these favorable effects of the total antioxidant capacity may be mediated by vitamin C, vitamin E and its isomers, selenium, carotenoids, isoflavones, flavonoids and proanthocyanidins [44, 45]. In this regard, there are numerous studies which have indicated the beneficial effects of high-antioxidant foods (for example, fruits, vegetables, olive oil, nuts and tea) on the obesity, insulin resistance, glucose homeostasis and lipid profiles [46, 47]. These beneficial effects of antioxidants on the metabolic profile may be partly attributed to other activities of antioxidants such as regulation of metabolic pathways in brown adipose tissue and increase thermogenesis, suppression of adipogenesis and induction of catabolism in adipose tissue [48].

### Strengths and weaknesses

As far as we know, this is the first study to examine the interaction of CARTPT rs2239670 genotypes with dietary NEAC on the metabolic factors in obese subjects and identifying these gene–diet interactions may provide the best personalized dietary advice for high-risk participants according to their genetic makeup to decrease the heavy burden of obesity and its-related chronic diseases. However, the present study has certain limitations that need to be noted. Firstly, since this is a cross-sectional study, ascertained causality cannot be argued but it helps to generate hypotheses that can be examined by prospective cohort or other studies. Secondly, a rather small sample size of our study may not cover statistical power for analyzing the interaction effect. So, the results of our study must be interpreted warily and require replication and confirmation in larger and different populations. Thirdly, the present study was limited to the assessment of only single polymorphism from a single gene, while there are multiple well-known genes that have been implicated in the pathogenesis of obesity and its related consequences. Fourthly, our results may not necessarily be extrapolated to the general population as this study was carried out among a population from Tabriz with different cultures and lifestyle factors. Fifthly, despite adjustment for several confounders in the analyses, residual confounding by other unmeasured factors was inevitable. Lastly, under-reporting of dietary intake, as a potential bias, is common among obese individuals which may cause underestimation of the true effect [49]. Thus, we excluded the extreme-energy reporters from analysis.

## Conclusion

In conclusion, our finding showed a statistically significant gene–diet interaction between the CARTPT rs2239670 and compliance with healthy and good quality diet rich in antioxidants in relation to obesity and related metabolic phenotypes; high intake of NEAC by minor allele carriers attenuated genetic association with BMI and FM%; however, high compliance with these indices could not affect genetic predisposition to blood sugar abnormalities. Further studies are warranted to confirm our results, which may be of important in public health.

## Data Availability

Data of the current research will be available with a reasonable request from the corresponding author.

## Abbreviations

NEAC: Non-enzymatic antioxidant capacity
CARTPT: Cocaine- and amphetamine-regulated transcript prepropeptide
FFQ: Food frequency questionnaire
ORAC: Oxygen radical absorbance capacity
FRAP: Ferric reducing antioxidant power
TRAP: Total radical-trapping antioxidant parameter
TEAC: Trolox equivalent antioxidant capacity
CVDs: Cardiovascular diseases
ROS: Reactive oxygen species
MetS: Metabolic syndromes
